# Advances in Graphene-Based Flame-Retardant for Polystyrene Applications: Synthesis, Mechanisms, and Future Perspectives

**DOI:** 10.3390/polym17131811

**Published:** 2025-06-29

**Authors:** Mutawakkil Isah, Farrukh Shehzad, Mamdouh A. Al-Harthi

**Affiliations:** 1 Department of Chemical Engineering, King Fahd University of Petroleum and Minerals, Dhahran 31261, Saudi Arabia; g202115810@kfupm.edu.sa; 2 Interdisciplinary Research Center for Refining & Advanced Chemicals, King Fahd University of Petroleum & Minerals, Dhahran 31261, Saudi Arabia

**Keywords:** graphene, functionalized graphene, polystyrene, flame retardants, thermal stability, fire resistance

## Abstract

The growing demand for fire-safe, sustainable materials has driven extensive research into advanced flame retardants particularly polystyrene (PS), a widely utilized yet inherently flammable polymer. Graphene-derived materials are considered effective flame retardants owing to their higher thermal stability, char-formation, and gas barrier properties. However, despite these advantages, challenges such as agglomeration, high thermal conductivity, poor interfacial compatibility, and processing limitations hinder their full-scale adoption in building insulation and other applications. This review presents an in-depth analysis of recent progress in graphene-enhanced flame-retardant systems for polystyrene applications, focusing on synthesis methods, flame-retardant mechanisms, and material performance. It also discusses strategies to address these challenges, such as surface functionalization, hybrid flame-retardant formulations, optimized graphene loading, and improved dispersion techniques. Furthermore, future research directions are proposed to enhance the effectiveness and commercial viability of graphene-based flame-retardant polystyrene composites. Overcoming these challenges is essential for high-performance, eco-friendly, flame-retardant materials on a larger scale.

## 1. Introduction

Polymer-based materials are extensively utilized across various industrial and domestic applications due to their exceptional properties, widespread availability, and ease of processing [1,2]. As a result, they hold a large share of market products and largely replaced inorganic materials in many fields of application [3,4]. PS is extensively utilized in diverse applications, construction materials, automotive and transportation systems, and building insulations, due to its beneficial characteristics, including low electrical and thermal conductivity, resistance to corrosion, and lightweight structure [5]. However, PS has intrinsic limitations stemming from the organic composition of these polymer materials. This makes them highly flammable and a considerable fire risk, restricting their use in areas such as electronics, transportation, structural components, and other fire-sensitive applications [6,7], where minimal flammability and high thermal stability are crucial [8,9]. To overcome this drawback, flame-retardant additives have been incorporated to reduce flammability and enhance their thermal stability [10,11]. Incorporating nanofillers into the PS matrix is an effective approach to enhancing flame retardancy [12]. Hence, integrating flame-retardant materials is crucial to enable the safe application of PS-based materials in diverse fields [13,14]. Various flame-retardant materials, such as halogenated compounds, have been widely used [15]. As noted earlier, halogen-based flame retardants are highly effective in providing flame resistance. However, their combustion releases toxic gases, posing serious risks to human health and the environment. Consequently, growing concerns about environmental sustainability have driven a shift toward halogen-free flame retardants, which are increasingly replacing traditional halogenated compounds [16,17,18].

Graphene consists of a single two-dimensional (2D) sheet of carbon atoms arranged in an sp^2^-hybridized structure, and is an advanced and environmentally friendly material. Its high thermal stability, flame retardancy, low required dosage, and strong environmental compatibility make it promising for various engineering applications [19,20,21]. Numerous studies demonstrate that graphene and its derivatives can impact the thermal conductivity, pyrolysis, heat absorption, viscosity, and dripping behavior of polymers. These properties make them highly effective flame retardants, as they enhance the thermal stability of polymers, prolong ignition time, suppress flame spread, and lower the heat release rate [22]. Incorporating graphene sheets into polymer matrices resulted in a unique category of polymer nanocomposites, valued for their enhanced thermal, electrical, and mechanical performance across a wide range of applications [23]. Additionally, the new polymer nanocomposites exhibit flame-retardant properties, which are attributed to the graphene sheets’ ability to form a compact char layer. This layer acts as a physical barrier, limiting the release of combustible degradation products and thereby slowing the polymer degradation process [24].

**Figure 1:**
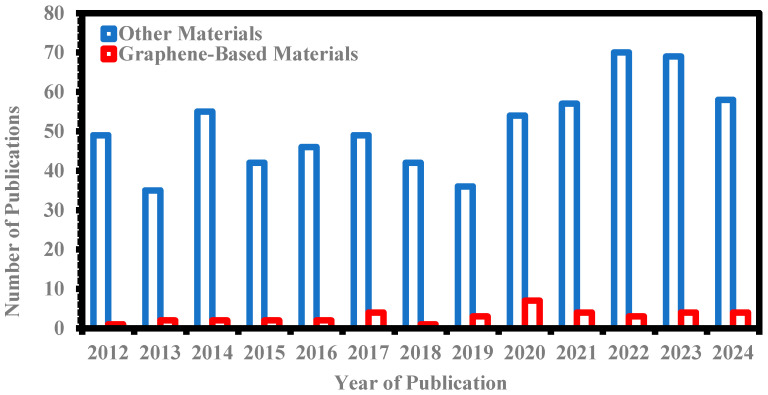
Number of published articles on polystyrene flame retardants based on graphene-based and other materials from 2012 to August 2024, according to the Scopus database.

Since their discovery, extensive research has been conducted on graphene sheets as flame-retardant fillers in various polymers. However, comparatively fewer studies focused on the application of graphene-based materials specifically in PS, as highlighted in Figure 1. The figure reveals a clear imbalance in research output, where other flame-retardant materials for PS received far more attention than graphene-based systems, despite the latter’s excellent thermal stability, high flame resistance, and barrier properties. This underrepresentation suggests a significant research gap, likely due to challenges such as dispersion difficulties, processing complexity, and material cost. Given polystyrene’s wide use in construction and transportation areas where flame retardancy is critical, further research in this domain is essential. This review presents a comprehensive analysis of studies on graphene and its derivatives as flame-retardant fillers in polymer matrices, with a focus on their classification, flame-retardant mechanisms, and emerging trends. Special emphasis is placed on the promising role of functionalized graphene in overcoming the limitations of PS by enhancing thermal stability, flame resistance, and compatibility within the matrix, thus offering a pathway for safer and more advanced PS-based materials. Although several review papers addressed the use of graphene-based materials as flame retardants in polymers [8,25,26,27], most of those have a broad approach, i.e., covering diverse polymer systems or multifunctional applications, with a limited emphasis on PS. However, this review provides a focused overview of functionalized graphene systems specifically for PS, with emphasis on the effect synthesis and surface modifications on the flame retardancy. It also explores flame retardancy mechanisms specific to PS, offers quantitative comparisons using key metrics such as LOI and PHRR, and outlines future directions tailored to PS applications. Moreover, to ensure relevance, this review primarily covers studies published between 2014 and 2025.

## 2. Fundamentals of Flame Retardancy in Polymers

### 2.1. Combustion Mechanism

**Scheme 1:**
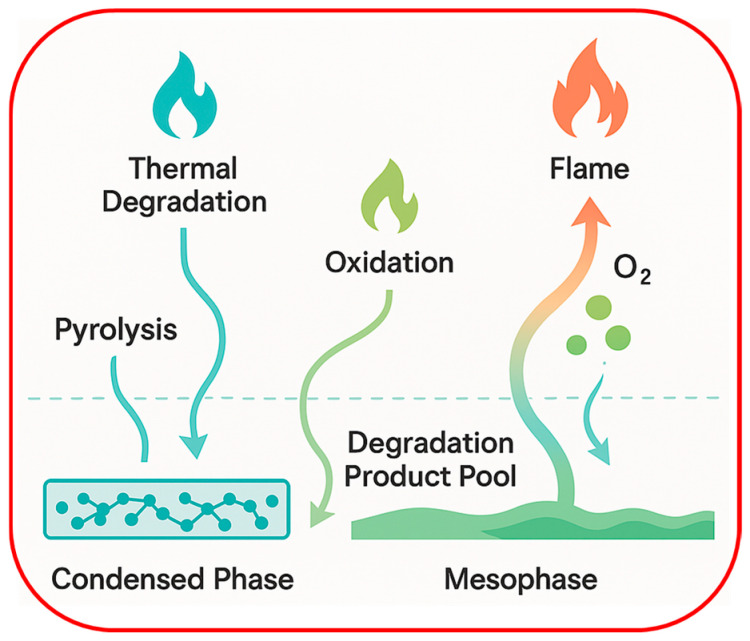
Polymer combustion pathways across the condensed, mesophase, and gas phases. The diagram shows how polymer combustion progresses through condensed, mesophase, and gas phases. Heat induces pyrolysis, generating volatiles that ignite in the gas phase with oxygen. The resulting flame sustains combustion, while melted polymer forms flammable pools that pose secondary fire risks.

The combustion of polymers can take place in the condensed phase, gas phase, and mesophase. Upon exposure to an external heat source, polymers undergo pyrolysis, generating increasing amounts of flammable volatiles that transition into the gas phase as indicated in Scheme 1. These volatiles combine with atmospheric oxygen, forming a combustible mixture that ignites and sustains the flame. The heat released during combustion is then transferred back to the polymer’s condensed phase, further propagating the burning process. Additionally, many polymers melt and flow during combustion, leading to the formation of flammable degradation product pools, which pose a significant secondary fire hazard by potentially igniting or spreading flames to nearby materials [28,29].

When polymer materials are heated to a specific temperature, they spontaneously generate volatile combustible substances. Combustion occurs when these substances accumulate in the air to a critical concentration. The combustion process of polymer materials involves two stages: thermal degradation and oxidation-induced combustion. This process includes the degradation and decomposition of polymers within the condensed phase, along with the diffusion of the resulting byproducts across both the solid and gas phases. Concurrently, these degraded compounds can interact with air, initiating oxidation reactions and propagating a chain reaction that sustains combustion [30].

### 2.2. Flame-Retardant Mechanism

The mechanism of flame retardancy works through several key processes that collectively reduce flammability, inhibit flame spread, and enhance material safety. These mechanisms include the endothermic effect, where flame retardants absorb substantial heat during combustion, initiating a strong endothermic reaction that lowers the surface temperature, minimizes the smoke production, and delays the ignition. Additionally, the covering effect involves the creation of a stable foam layer on the surface of the flammable material, acting as a physical barrier that shields it from oxygen and suppresses the emission of flammable gases, which results in limiting fire progression [31,32]. On the other hand, the radical scavenging mechanism is essential in breaking the combustion chain reaction by neutralizing free radicals within the flame zone, thereby lowering flame intensity and ultimately extinguishing the fire. Another key mechanism involves the release of non-combustible gases; at elevated temperatures, flame retardants decompose to emit inert gases that dilute flammable vapors and help suppress the fire. In the condensed phase, the formation of protective carbon layers on the surface of the material slows down the thermal degradation, limits the release of flammable gases, and improves thermal insulation [33,34].

These mechanisms may also involve altering the polymer’s physical properties, such as reducing melting and pyrolysis temperatures, interrupting heat released during combustion, or increasing polymer heat resistance through crosslinking or the incorporation of aromatic structures. Together, these processes provide a multi-faceted approach to flame retardancy, ensuring improved fire resistance, reduced heat release, and enhanced safety for polymer-based materials [35].

Graphene-based flame retardants operate through a synergistic combination of mechanisms at the molecular level to reduce the flammability of PS. π–π stacking interactions between graphene sheets and PS chains promote the formation of robust, carbon-rich char layers that physically shield the material from heat and oxygen [36]. These protective char layers, often reinforced by the graphene’s structure, are pivotal in the condensed-phase flame-retardant action. In parallel, graphene additives can interfere with the flame chemistry by trapping free radicals (especially when used in concert with N or P functional groups) and by slowing the diffusion of combustible fragments into the flame zone [37]. The graphene-derived char and its labyrinthine layered structure act as an insulating barrier, drastically reducing heat release and slowing polymer decomposition [36]. Its high thermal conductivity aids in distributing heat, which helps prevent runaway decomposition, although excessive connectivity can counteract the barrier effect, thus optimal dispersion is key [27]. Additionally, the inherent chemistry of graphene (especially GO/rGO) provides catalytic sites that foster char-forming reactions in PS, effectively converting more of the polymer into char rather than flammable volatiles [36]. Taken together, these mechanisms result in a marked enhancement of fire resistance: graphene-loaded PS composites exhibit lower peak heat release rates, higher char residues, reduced smoke and toxic gas output, and in many cases self-extinguishing behavior [38]. 

Several examples of literature reviewed herein underscore that the molecular design of graphene-based flame retardants—tuning their surface chemistry for π–π interactions, combining them with radical scavengers, and ensuring good dispersion is vital to harness their full fire-suppressing potential in polystyrene. Ongoing research is expanding on these principles, exploring hybrids (e.g., graphene with layered double hydroxides, or graphene with intumescent systems) to further leverage catalytic char formation and gas-phase radical quenching [27]. Generally, graphene and its derivatives represent a multifaceted approach to flame retardation of PS, simultaneously attacking the problem at the chemical (radical) and physical (barrier) levels. This integrative mechanism, forming a heat-resistant char shield and interrupting combustion kinetics, highlights graphene-based nanomaterials as a highly effective and promising class of flame retardants for polystyrene and other aromatic polymer systems.

### 2.3. Conventional Flame Retardancy Tests

#### 2.3.1. Limiting Oxygen Index (LOI)

**Figure 2:**
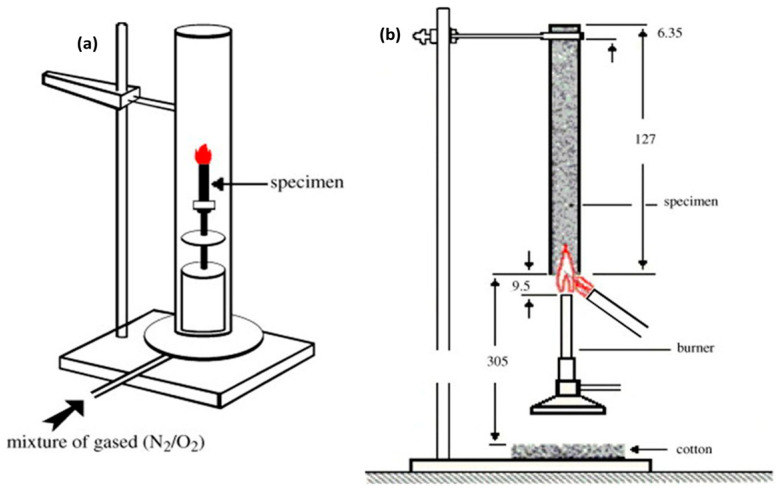
Flammability test. (**a**) LOI diagrammatic representation of the instrument and (**b**) the procedure for the UL 94 vertical flame test. Reproduced with permission from [43], MDPI, © 2020 licensed under CC BY 4.0 [43].

The limiting oxygen index refers to the lowest concentration of oxygen in an oxygen-nitrogen mixture required to sustain a flame for at least 3 min or to consume 50 mm of the test sample. As specified by the ISO 4589-2 [39] standard, an 80 mm × 10 mm × 4 mm sample is positioned vertically within a glass chimney, and its top is ignited using a burner. ASTM D2863 [40], corresponding to ISO 4589-2 [39], establishes the standards for LOI measurement. A lower LOI value indicates that a material is more flammable, as it can ignite in environments with less oxygen. For a material to be considered flame-retardant, its LOI should exceed 21% the oxygen level in the atmosphere. Materials with LOI values above this threshold, typically ranging from 22% to 30%, are classified as fire-retardant [41]. Polystyrene has an LOI of 18%, which makes it highly flammable. Therefore, there is a need to improve its LOI to improve its thermal stability and flame resistance. LOI testing is widely recognized as a key screening and quality control technique for developing flame-retardant polymers, due to its cost-effectiveness and the minimal sample size required. However, this method is limited in its ability to evaluate the actual fire resistance of materials, as it involves low heat input and operates under artificially high oxygen concentrations [42]. LOI results are reliable; however, there is no direct correlation between LOI testing and UL 94 flame tests. The LOI values do not exhibit a linear relationship with UL 94 ratings and cannot be used interchangeably. Figure 2 presents a schematic representation of the LOI and UL 94 test methods.

#### 2.3.2. Cone Calorimetry

Conical calorimetry (CC) is a widely recognized small-scale technique for evaluating the combustion behavior of polymeric materials conducted in accordance with international standards (ISO 5660) [44]. During this test, the sample is exposed to a conical radiant electric heater and ignited with an electric spark. The heat release rate (HRR) is determined by measuring the gas flow and oxygen levels, with the peak heat release rate (PHRR) serving as a critical indicator of the material’s fire resistance. The total heat release (THR) is determined by integrating the HRR over time. Additionally, conical calorimetry measures various combustion parameters, such as flameout time (TOF), time to ignition (TTI), effective heat of combustion (EHC), mass loss, carbon monoxide (CO) and carbon dioxide (CO_2_) emissions, and total smoke release (TSR). This method provides comprehensive fire behavior data, making it a valuable approach for replicating material flammability in realistic fire scenarios [45]. The cone calorimeter test operates on the principle of measuring the decrease in oxygen concentration in the combustion gases of a sample subjected to a specified heat flux ranging from 10 to 100 kW/m^2^. As per standard protocols, each gram of oxygen consumed during combustion corresponds to the release of 13.1 kJ of heat, a constant value regardless of the material tested [46].

Currently, the cone calorimetry test is considered as the most reliable method for stimulating real fire conditions in a laboratory setting utilizing medium-sized samples to study the combustion behavior materials. By evaluating the smoke and heat parameters obtained from the cone calorimeter test, the flammability of materials can be quantitatively determined using the following key indicators:

1. *TTI* (s): Under fixed irradiation intensity and sample thickness, a longer ignition time indicates greater resistance to ignition. However, in flame-retardant polymers, the presence of flame retardants may cause premature decomposition, reducing the TTI. Hence, a shorter TTI does not necessarily indicate reduced flame retardancy.
2. *HRR* (kW/m^2^): The heat release rate quantifies the energy emitted per unit time and surface area during combustion, typically expressed in kilowatts per square meter. The peak heat release rate (PHRR), representing the maximum HRR observed, serves as a critical metric for assessing a material’s fire performance.
3. *THR* (kJ/m^2^): The total heat released per unit area during combustion is calculated by integrating the HRR over the duration of the burning process.
4. *Fire growth rate* (*FGR*, kW/(s·m^2^)): Defined as FGR = PHRR/tPHRR, where tPHRR is the time required to reach the peak HRR. A lower FGR value indicates better fire resistance.
5. *Mass loss rate* (*MLR*, g/s): Represents the rate at which the material loses mass during combustion.
6. *Smoke production rate* (*SPR*, m^2^/s) *and total smoke production* (*TSP*, m^2^): These parameters indicate the extent of combustion and the amount of smoke generated, providing insights into material flammability and smoke hazards.

#### 2.3.3. Tests for Flammability of Plastic Materials UL-94

Melting and dripping are the primary factors that contribute to fire propagation during polystyrene combustion. Molten polymers can facilitate flame spread, thereby accelerating fire propagation. To evaluate and control the use of flame-retardant polymers, it is essential to conduct vertical and horizontal flammability tests [47]. Developed by Underwriters Laboratories, the UL 94 test series is a widely adopted method for assessing the flammability of plastic materials used in appliances and equipment components. The UL 94 vertical burning test serves as the standard method for determining flammability and flame spread characteristics of plastics. Polystyrene composites are primarily evaluated using the UL 94 vertical burning test, focusing on classifications V-0, V-1, or receiving no rating, with detailed criteria outlined in Table 1. A specimen with consistent and predetermined dimensions is exposed to a 50 W blue flame, measuring 20 mm in height. The lower section of the sample is subjected to the flame for 10 s before being withdrawn. In the event of ignition, the duration required for self-extinguishment is recorded. Following flame extinction, the procedure is repeated by reapplying the flame for an additional 10 s, with the process conducted a total of five times [48].

The time taken for the flame to extinguish is referred to as the residual flame time (T1). After extinguishment, the lower end of the specimen is subjected to a second 10 s flame exposure. The time taken for the flame to self-extinguish during this second exposure is recorded as the residual flame time (T2), while the duration for the glow to completely disappear is designated as the residual glow time (T3). In accordance with IEC 60695-11-10 [49], five identical samples are tested in parallel during each experimental run [50]. For a material to achieve a V-0 rating, the flame must extinguish within 10 s on a vertically oriented specimen, and any dripping particles must not be ignited. A V-1 rating is assigned if the flame self-extinguishes within 30 s, with non-burning drips permitted. In contrast, a V-2 rating is given when the flame also self-extinguishes within 30 s, but in this case, flaming droplets are allowed [51].

**Figure 3:**
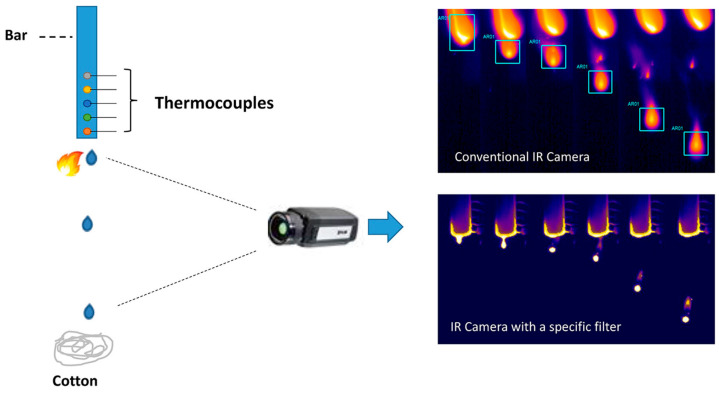
A thermocouple-embedded bar and an infrared camera with a specialized filter are used to measure the thermal gradients of the burning polymer and its droplets. Reprinted with permission from [52], Elsevier, Copyright (2018).

Figure 3 illustrates an enhanced UL 94 testing setup, equipped with integrated thermocouples and infrared cameras fitted with specialized filters. This configuration facilitates the measurement of the heat gradient within the specimen and the surface temperature of the dripping polymer, allowing for advanced data collection [52]. A drawback of this test is its sensitivity to the specimen’s thickness, which affects the results. Additionally, it does not provide an analysis of the material’s inherent properties [53]. Recent advancements facilitated the development of numerical modeling for the UL 94 burn test, demonstrating promising outcomes. This method allows for the prediction of flame spread and HRR in complex polymer systems [54].

## 3. Graphene as a Flame Retardant: An Overview

### 3.1. Unique Properties of Graphene

**Figure 4:**
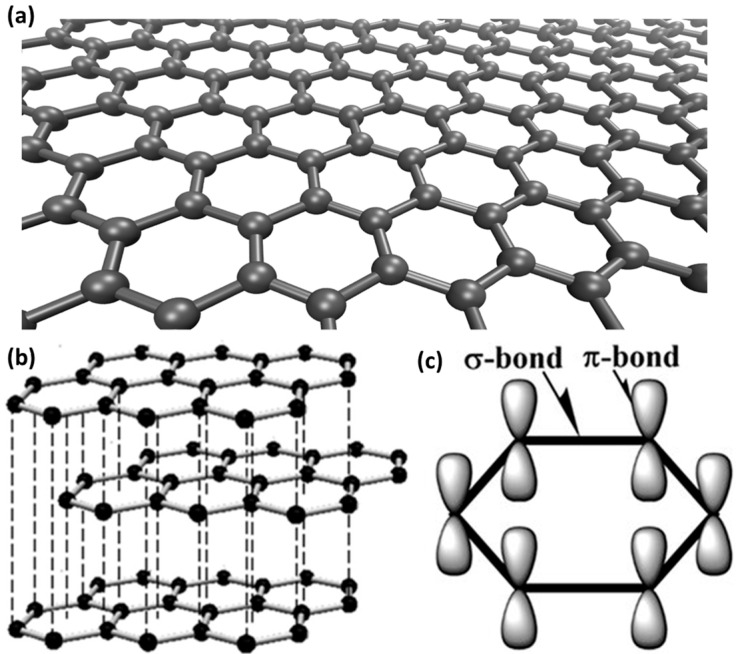
(**a**) The hexagonal arrangement of graphene’s lattice, reprinted with permission from [61], MDPI, © 2019 by the authors; licensed under CC BY 4.0. (**b**) The stacking of graphene layers, reproduced with permission from [62], the Royal Society of Chemistry, 2022. (**c**) Diagrammatic representation of the distribution of π orbitals and in-plane σ bonds in graphene layers, reprinted with permission from [63], Elsevier, Copyright (2022).

Graphene is a carbon-based nanomaterial consisting of two-dimensional, single-atom-thick layers of carbon atoms bonded in an sp^2^ configuration and arranged in a tightly packed honeycomb lattice [55,56]. The structure of graphene resembles a series of interconnected benzene rings, with carbon atoms taking the place of hydrogen atoms, as shown in Figure 4a. This configuration gives graphene a hydrophobic nature, owing to the lack of oxygen-bearing functional groups [57]. Graphene is a carbon allotrope made up of a single layer of sp^2^-bonded carbon atoms, each separated by a bond length of 0.142 nm. When multiple graphene layers are stacked together, they form graphite, characterized by an interlayer distance of about 0.335 nm [58]. This stacking configuration gives rise to the three-dimensional structure of graphite (Figure 4b), whereas graphene remains a two-dimensional, single-atom-thick material exhibiting sp^2^ hybridization. In this structure, each carbon atom forms three in-plane σ bonds and one π orbital oriented perpendicular to the plane (Figure 4c). The strong in-plane σ bonds establish a rigid hexagonal framework, while the out-of-plane π bonds facilitate interactions between graphene layers. Structural variations in graphene mainly result from the absence of one or more sp^2^-hybridized carbon atoms or the incorporation of atoms with sp^3^ hybridization [59,60].

In recent years, single-layered sp^2^-bonded carbon allotropes with dimensionalities ranging from 0 to 3D, such as fullerenes, carbon nanotubes, graphite, and graphene, have been incorporated into various polymer matrices. These materials are valued for their remarkable thermal stability, mechanical strength, electrical conductivity, and low weight, enhancing the performance of composite polymers across multiple applications [64]. Graphene’s distinctive properties stem from the covalent bonding of carbon atoms within its plane, where each carbon atom establishes three in-plane σ bonds with adjacent atoms and one out-of-plane π bond. This continuous network of sp^2^-hybridized carbon atoms gives graphene its outstanding electrical, mechanical, and thermal performance [65]. Graphene has a tensile strength of approximately 125 GPa and an elastic modulus of 1.1 TPa, making it about 100 times stronger than steel. This extraordinary strength results from its robust sp^2^-bonded carbon structure, which enables graphene to withstand considerable force without breaking [66]. Graphene exhibits higher thermal conductivity, around 5 × 103 W/mK, making it approximately ten times more conductive than copper (401 W/mK). Its electron mobility is extremely high, with a conductivity of 106 S/m and a sheet resistance of 31 Ω/sq, resulting in electron mobility of about 2 × 105 cm^2^/Vs, which is 140 times greater than that of silicon. This exceptional mobility arises from graphene’s sp^2^ hybridization, which contributes an additional electron to the π bond; these delocalized π electrons are mobile even at room temperature, leading to graphene’s high electrical conductivity [67].

**Figure 5:**
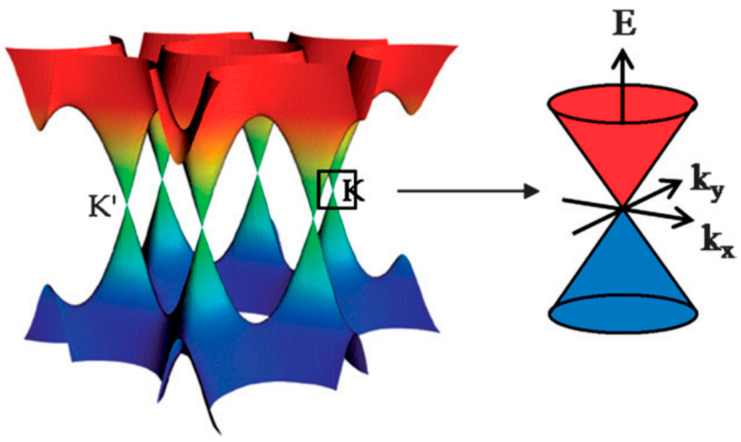
Electronic band structure of single-layer graphene, the conduction band and valence band overlapped to one another showing zero band gap, reprinted with permission from [69], =the Royal Society of Chemistry.

Graphene is inherently a semimetal, with an electronic structure that mirrors the arrangement of benzene rings. It contains six π-orbitals three of which are filled bonding orbitals, while the other three are unoccupied antibonding orbitals separated by a small bandgap. The fusion of benzene rings results in limited overlap between the valence and conduction bands, allowing electrons at the top of the valence band to move into the conduction band without the need for thermal excitation (see Figure 5). This unique feature contributes to graphene’s remarkable electrical conductivity, as electrons can flow freely across the material without requiring energy input to overcome a significant bandgap [68,69,70,71]. Graphene’s specific surface area is influenced by the degree of layer stacking. However, when graphene is functionalized with other compounds, this stacking is minimized, leading to an enhancement in its effective surface area. By reducing stacking and promoting better dispersion, modifications can enhance graphene’s surface area, making it more accessible for applications such as catalysis and energy storage. The increased surface area improves the material’s performance by providing more active sites for chemical reactions or interactions with other materials [72]. In recent years, graphene garnered widespread interest from researchers, owing to its remarkable electronic, thermal, mechanical, optical, and magnetic characteristics, as well as its extensive surface area [73,74,75].

### 3.2. Various Synthesis Methods for Graphene Sheets

Various techniques have been utilized in recent years for the synthesis of graphene. The extraction process differs based on the desired purity and the targeted product [76]. Since the discovery of graphene in 2004, various methods have been developed for the fabrication of thin graphene layers and films [77]. The selection between top-down and bottom-up synthesis methods depends on factors such as the layers number, thickness, and the properties and average dimensions of the resulting graphene materials. In the top-down approach, graphene sheets are obtained by exfoliating or separating graphite and its derivatives, such as graphite oxide (GO). Table 2 presents an overview of both top-down and bottom-up synthesis techniques, highlighting their respective benefits and limitations [78].

The synthesis method of graphene plays a critical role in determining its effectiveness in flame-retardant applications. Each method not only influences the physical characteristics of the graphene produced, such as thickness, lateral size, and purity, but also affects its chemical properties, such as the presence of functional groups, which directly impact its compatibility with polymer matrices and its ability to promote char formation during combustion.

**Figure 6:**
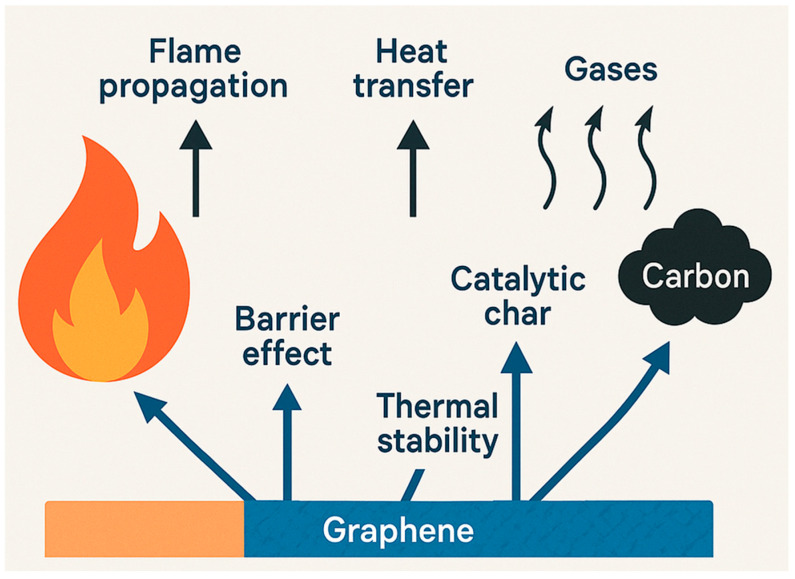
The role of graphene in flame-retardant materials.

Among the available methods, electrochemical exfoliation emerged as one of the most suitable approaches for flame-retardant applications. This technique involves applying an electric field to graphite electrodes immersed in an electrolyte solution, resulting in the separation of graphene layers. A key advantage of this method is its ability to introduce functional groups, such as hydroxyl and carboxyl groups, onto the graphene surface. These groups significantly enhance the dispersion of graphene within polymer matrices and facilitate strong interfacial interactions, which are cru-cial for forming a continuous char barrier during combustion. This char acts as a physical shield, slowing heat transfer and volatile gas release, thereby improving the flame-retardant performance of the composite. Figure 6 shows the roles of graphene in flame-retardant composites through char formation and gas barrier effect. Electro-chemically exfoliated graphene has been shown to reduce PHRR and THR in polymers such as epoxy and polystyrene, confirming its superior performance in fire safety ap-plications [22].

On the other hand, CVD produces high-purity, large-area graphene with minimal structural defects. While this makes CVD graphene ideal for electronic applications, its lack of functional groups reduces its effectiveness in flame-retardant formulations. The pristine nature of CVD graphene limits its dispersion within the polymer and its ability to interact chemically with the matrix. Furthermore, the high temperature and cost associated with CVD make it less practical for bulk flame-retardant production [79].

Reduction in GO is another common method for producing graphene, wherein oxidized graphite is chemically or thermally reduced. This approach results in graphene with residual oxygen-containing groups that can improve flame retardancy by aiding char formation. However, the presence of these oxygen groups can also compromise thermal stability, and the process may introduce impurities, limiting its performance in demanding fire safety applications [80].

Considering all factors, including dispersion ability, chemical functionality, cost, and scalability, electrochemical exfoliation is the most favorable method for synthesizing graphene intended for flame-retardant materials. It combines high yield and moderate production costs with functionalization potential, making it effective for enhancing the thermal stability and flame resistance of polymer composites.

### 3.3. Graphene as Flame-Retardant Material

Graphene and its derivatives, known for their exceptional heat resistance, demonstrated promising flame-retardant properties, effectively mitigating the thermal hazards of polymer matrices to a certain extent [81,82,83]. Incorporating a small amount of graphene sheets into the polymer matrix improves the thermal stability and mechanical performance of polymer nanocomposites [32,84,85]. Studies indicated that incorporating 3 wt% graphene sheets into polyvinyl alcohol (PVA) significantly reduces its flammability properties [24]. Furthermore, the flammability parameters measured using a cone calorimeter confirm the significant enhancement in flame resistance of the resulting polymer nanocomposite. Consequently, a noticeable decrease was observed in the PHRR, TTI, LOI, THR, and UL-94, as shown in Table 3. However, when an equivalent mass fraction of clay layers (Na-MMT) and multiwalled carbon nanotubes (MWNT) was incorporated into PVA, the resulting reduction in flammability of the polymer nanocomposites was less effective compared to those containing graphene. This enhanced flame-retardant performance in graphene-based nanocomposites is attributed to the formation of a compact and dense char layer by graphene sheets during combustion. This char barrier effectively shields the polymer degradation zone from the high-temperature flame region [24,86,87]. Consequently, the HRR curves demonstrate the effectiveness of graphene sheets as flame-retardant additives, showing superior performance in flame suppression compared to clay layers and MWNT-based nanofillers. The improved flame-retardant behavior of graphene and/or graphene oxide layers has been further observed by numerous studies involving a range of polymeric and textile materials [88,89,90,91].

## 4. Functionalization of Graphene for Enhanced Flame Retardancy

The most prominent and extensively used method for synthesizing GO is the one introduced by Hummers and Offeman [120]. This method provides three key advantages compared to other techniques. Firstly, the reaction concludes within a few hours. Secondly, substituting potassium chlorate with potassium permanganate improves reaction safety. Lastly, the use of sodium nitrate minimizes the generation of acid mist. Despite its advantages, this method has certain drawbacks. The oxidation process releases hazardous gases such as nitrogen dioxide and dinitrogen tetroxide. Additionally, removing sodium and nitrate ions from the wastewater generated during graphene oxide synthesis and purification poses a significant challenge [121].

**Figure 7:**
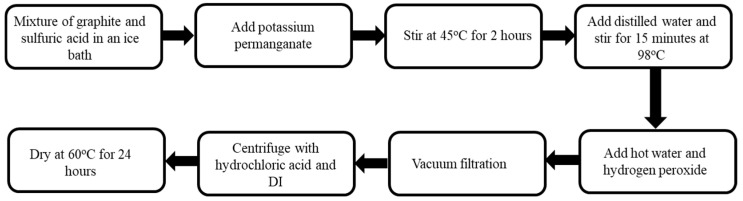
GO synthesis using the Hummers method conducted at 60 °C and 90 °C over a duration of 24 h, reprinted with permission from [121], MDPI, © 2022 by the authors under Creative Commons Attribution [121].

Earlier studies enhanced the Hummers method by removing sodium nitrate and increasing the concentration of potassium permanganate, thereby facilitating the reaction to proceed within a single reaction mixture [122,123,124]. This modification improves reaction efficiency, minimizes the emission of toxic gases, and introduces phosphoric acid into the reaction system. Previous research demonstrated that the combination of sulfuric acid and nitric acid in the Hummers method acts as “chemical scissors,” cutting through the graphene layers and allowing the oxidation solution to penetrate more effectively [125]. Potassium permanganate can fully intercalate graphite, forming graphite bisulfate [126]. This interaction allows potassium permanganate to efficiently penetrate the graphene layers, promoting the oxidation of graphite. Consequently, potassium permanganate assumes the role previously held by sodium nitrate, as a result, adding it to the reaction is unnecessary. A study by Nestor et al. demonstrates a straightforward and cost-effective synthesis route for producing GO using an environmentally friendly modified Hummers method [121]. This method exhibits high reproducibility and produces graphene oxide that is well-suited for subsequent reduction to reduced graphene oxide (rGO), as demonstrated in Figure 7.

**Figure 8:**
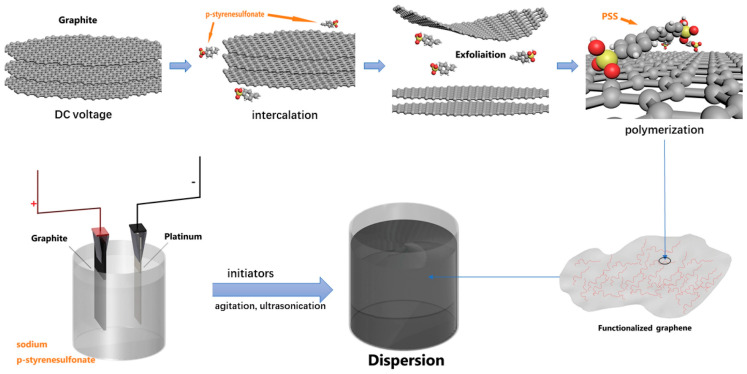
Illustration of the electrochemical process for exfoliating and functionalizing graphene, reprinted with permission from [111], Elsevier, Copyright (2020).

Zhao et al. [111] developed a novel technique for graphene functionalization involving the electrochemical synthesis of functionalized graphene nanosheets assisted by multifunctional p-styrene sulfonate. In this study, graphene was simultaneously exfoliated and functionalized via an electrochemical method conducted in a sodium p-styrene sulfonate solution. The p-styrene sulfonate served multiple roles: as an anionic exfoliating agent, a scavenger for hydroxyl and oxygen radicals through its vinyl groups and aromatic rings, and as a monomer for the formation of functional macromolecules—namely, polystyrene sulfonate (PSS). The process consisted of electrochemically exfoliating graphene from bulk graphite, followed by the free radical polymerization of p-styrene sulfonate. This strategy effectively yielded high-quality PSS@GNS, as indicated by a low ID/IG ratio of 0.17 in the Raman spectrum [111]. Figure 8 illustrates the electrochemical exfoliation mechanism. As anticipated, the intercalation of sodium p-styrene sulfonate into the interlayer spaces of graphite results in an expansion of the interlayer gaps. This increased spacing weakens the van der Waals interactions between the layers, thereby facilitating the exfoliation of graphene nanosheets.

A constant voltage of 15 V was supplied using a variable power source. The electrolyte was prepared by dissolving 5.0 g of sodium p-styrene sulfonate in 500 mL of deionized water. Graphite foil, acting as the anode, was submerged in the solution, while a copper rod, serving as the cathode, was placed 1 cm apart and aligned parallel to the foil. Electrochemical exfoliation of the graphite foil proceeded over a period of 6 h, resulting in the formation of graphene sheets. Polymerization of p-styrene sulfonate was then initiated by adding ammonium persulfate and ammonium sulfite. After 2 h of ultrasonic treatment, the resulting mixture was filtered under vacuum, and the collected filter cake was washed with deionized water to remove any remaining reagents. The product was freeze-dried, and the mass of the functionalized graphene nanosheets was measured using an analytical balance. For control purposes, graphene nanosheets were prepared under the same conditions, with sodium sulfate replacing sodium p-styrene sulfonate [111].

While both modified Hummers and electrochemical routes produce graphene derivatives with promising flame-retardant potential, they present distinct trade-offs in efficiency, safety, environmental impact, and material performance. The modified Hummers approach faces challenges related to the resultant chemical waste, scalability, and the partial removal of residual oxidating agents. On the other hand, the electrochemical method utilizing p-styrene sulfonate offers unique pros, e.g., simultaneous exfoliation and covalent functionalization, consequently providing superior dispersion and interfacial compatibility in polymer matrices. However, the electrochemical route’s primary limitations include higher energy consumption, precise voltage control, and uncertainties regarding large-scale reproducibility and cost optimization for industrial scale. A critical literature gap exists in comparative studies evaluating long-term stability, uniformity of functional groups, and the performance of resultant graphene composites under realistic fire scenarios (ASTM E1354) [127].

### Functionalized Graphene-Based Materials for Polystyrene Flame Retardancy

**Scheme 2:**
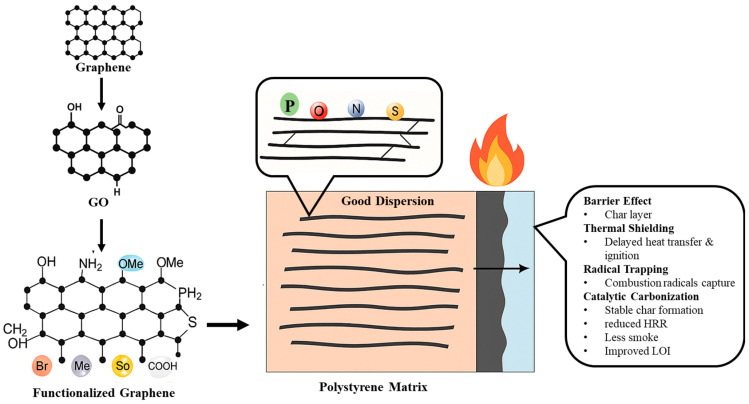
Illustration of flame-retardant mechanisms in polystyrene composites reinforced with functionalized graphene. The graphene nanosheets, chemically modified with elements such as phosphorus (P), oxygen (O), nitrogen (N), and sulfur (S), are uniformly dispersed within the polymer matrix. Under thermal exposure, these elements contribute synergistically to form a protective char layer, significantly enhancing flame resistance and thermal stability.

Sabet et al. synthesized single-layer graphene oxide from expanded graphite employing a modified version of the Hummers method [128]. In this study, GO was incorporated into PS to improve its thermal stability, enhance flame retardancy, and mitigate fire hazards. Cone calorimeter and limiting LOI tests indicated that the addition of GO resulted in a modest enhancement in flame resistance compared to pristine PS, as summarized in Table 3. Specifically, the incorporation of 3 wt.% GO improved the LOI value of PS from 18% to 18.8%. Dynamic mechanical analysis showed an improvement in both the storage modulus and the glass transition temperature (Tg) relative to neat PS. Furthermore, the PS/GO nanocomposites exhibited a reduction in the release of flammable volatiles and carbon monoxide, thereby contributing to improved fire safety. These findings underscore the potential of GO as an effective layered nanofiller to improve the flame-retardant properties of polystyrene composites [93]. The mechanism of flame-retardancy in PS composites is shown in Scheme 2.

Graphene and its derivatives exhibit exceptional thermal stability and act as effective flame retardants within the condensed phase. Their flame-retardant mechanism is primarily attributed to their two-dimensional layered architecture, which forms a protective barrier that significantly hinders heat transmission to the underlying polymer matrix undergoing thermal degradation [129,130,131]. However, a trade-off exists between the barrier effect and the inherently high thermal conductivity of rGO. Furthermore, the incorporation of graphene or its derivatives alone generally does not provide adequate flame retardancy in polymer systems. As a result, these materials are frequently functionalized and integrated with other flame-retardant agents to attain a synergistic enhancement in flame-retardant performance [132].

**Figure 9:**
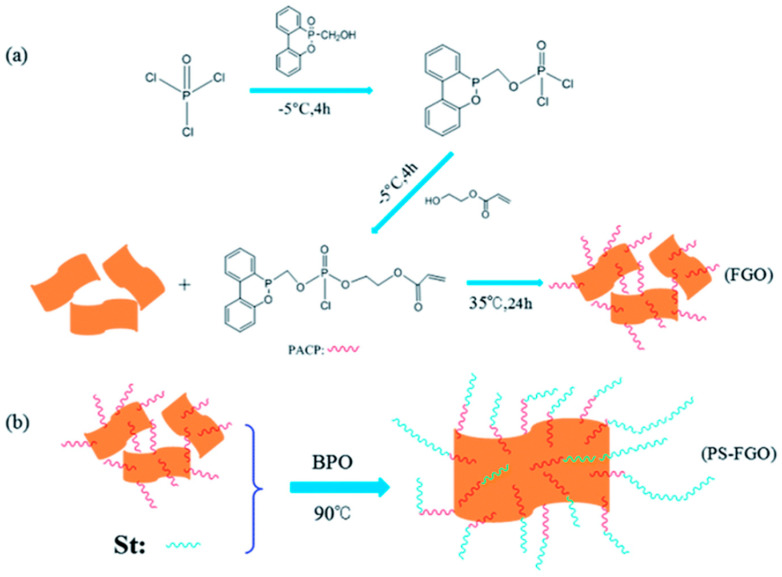
Synthesis paths of (**a**) FGO and (**b**) PS-FGO nanocomposites, reprinted with permission from [113], The Royal Society, © 2018 by the authors; licensed under CC BY 3.0 [113].

Dai et al. effectively functionalized graphene oxide by covalently grafting a newly developed phosphorus-containing flame retardant, [2-((6-oxidodibenzo[c,e][1,2]oxaphosphinin-6-yl)methoxy) acryloxyethylchlorophosphate], onto its surface. This functionalization aimed to enhance the flame retardancy of GO by introducing phosphorus-containing groups known for their fire-resistant properties (Figure 9). The synthesized FGO demonstrated hydrophobic properties and remained stability in polar solvents such as N,N-dimethylformamide (DMF). The presence of reactive vinyl groups in the PACP grafted onto FGO enabled copolymerization with styrene, resulting in the formation of polystyrene-FGO (PS-FGO) nanocomposites. Compared to pristine PS and PS-GO composites, the PS-FGO nanocomposites exhibited substantially enhanced fire resistance, thermal stability, and glass transition temperature. These enhancements are primarily attributed to the homogeneous dispersion of FGO within the PS matrix and the strong interfacial bonding between the FGO and the polymer chains [113].

**Figure 10:**
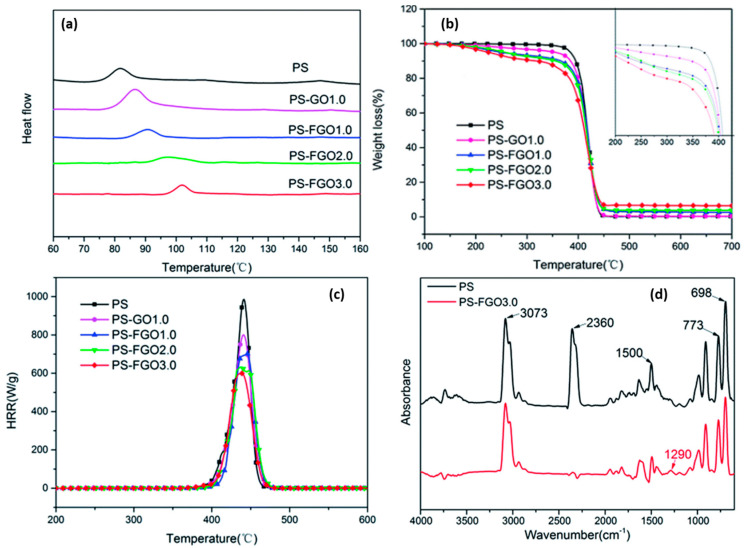
(**a**) DSC curves of PS and PS-GO/FGO nanocomposites, (**b**) TG curves of PS and PS-GO/FGO nanocomposites, (**c**) HRR curves of PS and PS-GO/FGO nanocomposites, and (**d**) FTIR spectra of pyrolysis products for PS and PS-FGO3.0 at MMLR, where GO stands for graphene oxide and FGO stands for a covalently functionalized graphene oxide, reprinted with permission from [113], The Royal Society, © 2018 by the authors; licensed under CC BY 3.0 [113].

The flammability performance of PS and PS-FGO nanocomposites were assessed through LOI and microscale combustion calorimetry (MCC) analyses. Neat PS showed a low LOI value of 18.1, reflecting its high flammability. However, the incorporation of increasing amounts of FGO significantly improved flame resistance, with the LOI value rising to 25.0 for the PS-FGO3.0 formulation, thereby demonstrating enhanced flame-retardant properties. MCC results reveal that the THR decreased as FGO content increased, with PS-FGO1.0 showing lower THR than PS-GO1.0, suggesting improved carbonization and cohesive char formation as shown in Table 3. Additionally, the heat release capacity (HRC) significantly decreased from 984 J g^−1^ k^−1^ (PS) to 599 J g^−1^ k^−1^ (PS-FGO3.0), a 39.1% reduction, due to lower MMLR and fewer combustible volatiles. These findings demonstrate that FGO enhances nonflammability by promoting char formation and reducing thermal degradation in PS nanocomposites. The operating temperature range of a polymer depends on its Tg, with a higher Tg enabling a broader range. The wrinkled surface of GO sheets enhances mechanical interlocking and restricts polymer chain mobility, leading to a 4 °C increase in Tg for PS-GO compared to pure PS. Notably, PS-FGO nanocomposites exhibit even higher Tg values, rising from 90 °C to 102 °C as the FGO content reaches 3 wt%, achieving a 20 °C increase as shown in Figure 10a. This improvement is due to the physical hindrance of polymer chain movement by the nanosheets and the covalent bonding of PACP in FGO with PS chains, which collectively constrain chain mobility more effectively.

Thermogravimetric analysis (TGA) was performed to study the thermal degradation of PS after incorporating GO and FGO. The results, shown in Figure 10b, reveal that pure PS undergoes a one-stage weight loss between 350 and 500 °C, decomposing into styrene monomers, oligomers, and volatiles, leaving minimal char residue. In contrast, PS-GO1.0 exhibits slightly lower thermal stability due to GO’s inherent instability [133]. PS-FGO1.0 shows further reduced stability, attributed to the breakdown of weak P–O–C bonds in FGO, which accelerates matrix degradation [134]. Unlike PS, FGO-containing samples display a two-stage weight loss between 150 and 350 °C, indicating that FGO significantly alters the decomposition process. Notably, char residue increases with FGO content, reaching 6.50 wt% for PS-FGO3.0 compared to 0.33 wt% for pure PS. This enhancement is due to GO nanosheets acting as physical barriers and FGO’s flame-retardant chains promoting char formation [135]. The higher char residue improves thermal stability by limiting oxygen and flammable gas transfer, reducing thermal conductivity, and increasing decomposition temperatures. The HRR curves and MCC data for PS and its nanocomposites are shown in Figure 10c. The THR of PS-FGO nanocomposites decrease as FGO content increases, with PS-FGO1.0 exhibiting a lower THR (23.4 kJ g^−1^) than PS-GO1.0. This demonstrates that FGO enhances carbonization during combustion, producing more cohesive char and fewer combustible volatiles, aligning with TGA results. The reduced THR indicates less heat release during combustion, lowering fire hazards. The HRC, a key flammability indicator, decreases significantly with FGO incorporation, dropping by 39.1% from 984 J g^−1^ K^−1^ (pure PS) to 599 J g^−1^ K^−1^ (PS-FGO3.0). This improvement stems from FGO’s ability to catalyze char formation, reduce maximum mass loss rates (MMLR), and limit combustible gas production [136,137]. Consequently, PS-FGO3.0, with the lowest MMLR, shows the best flame resistance due to reduced combustible volatiles and enhanced char formation.

FTIR spectra of pyrolysis products from PS and PS-FGO3.0 at MMLR were analyzed, as illustrated in Figure 10d. Key signals included CO_2_ (2360 cm^−1^), aromatic compounds (3073, 1500, 773, and 698 cm^−1^), and hydrocarbon groups (2980–2900 cm^−1^) [138,139]. Previous studies indicate that PS primarily decomposes into styrene, dimer, and trimer of phenyl alkenyl [140]. The DOPO moiety in FGO contributes to flame retardancy in the vapor phase. While the P–O group’s signal is obscured by overlapping with the C–O band at high temperatures, a new absorption band for P=O (1290 cm^−1^) [141], associated with PO_2_ in H_3_PO_2_ [142,143], appears in PS-FGO3.0. This volatile phosphorus compound acts as a flame quencher, suppressing gas-phase chain reactions during combustion and enhancing the flame retardancy of PS-FGO nanocomposites.

**Figure 11:**
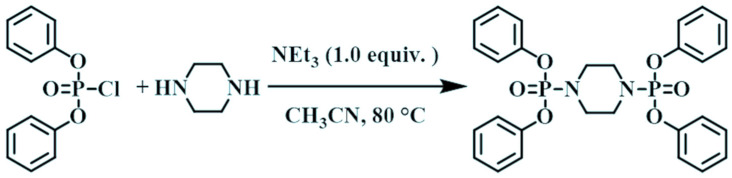
The synthetic pathway for the PON flame retardant, reprinted with permission from [105], Elsevier, Copyright (2023).

Wang et al. synthesized a novel phosphorus-nitrogen-based flame retardant (PON) via nucleophilic substitution of diphenyl phosphoryl chloride with piperazine as a precursor, as shown in Figure 11. When PS was blended with 10 wt% PON/10 wt% EG and 10 wt% PON/10 wt% GN, the LOI values significantly increased to 25.8% and 23.5%, respectively, compared to 18% for pure PS, demonstrating a significant enhancement in flame retardancy. The thermal stability of these intumescent flame-retardant systems was evaluated using TGA and DSC, confirming improvements in PS performance upon incorporation of the flame retardants. Moreover, notable reductions in PHRR, THR, and TSP by 74.6%, 24.4%, and 43.6%, respectively, were observed in comparison with pristine PS, as summarized in Table 3. The underlying flame-retardant mechanism was further elucidated through scanning electron microscopy (SEM), thermogravimetric infrared spectroscopy (TG-IR), and pyrolysis–gas chromatography/mass spectrometry (PY-GC/MS) analyses [110].

**Figure 12:**
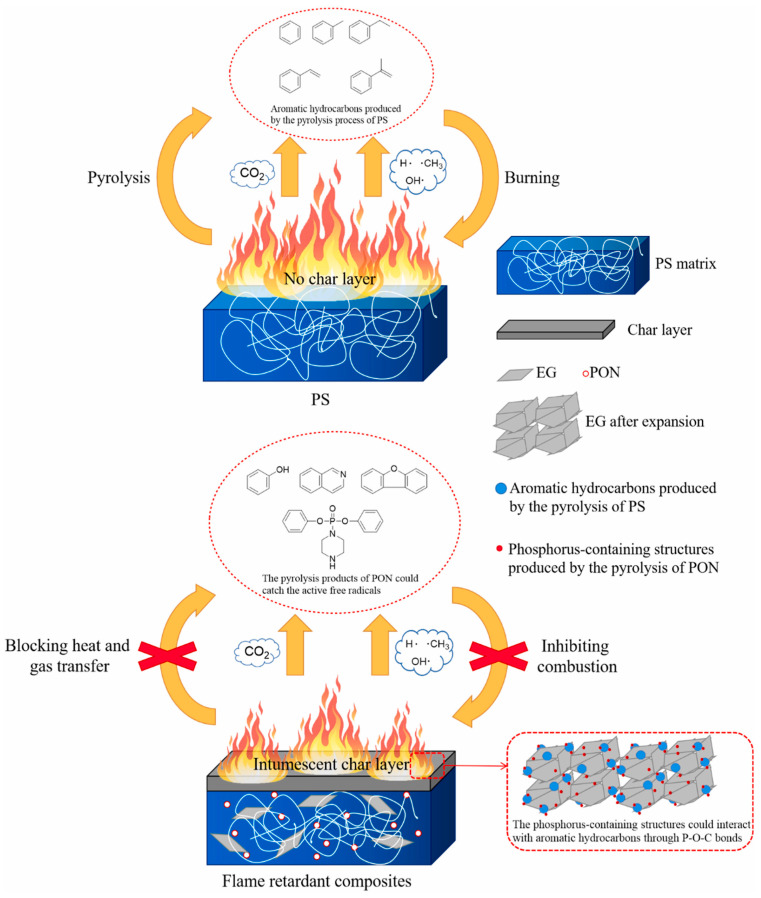
The proposed flame-retardant mechanism of PS/10%PON/10%EG composites, reprinted with permission from [110], Elsevier, Copyright (2023).

Based on the findings from SEM, TG-IR, and PY-GC/MS analyses, a flame-retardant mechanism has been proposed for the PS/10%PON/10%EG composite (Figure 12). In contrast to pure polystyrene, which undergoes vigorous combustion accompanied by dense, acrid black smoke and minimal residual char, its thermal degradation generates flammable gases and reactive free radicals (such as H, O, and OH). These species contribute to a positive feedback loop, intensifying the heat transfer to the polymer matrix, thereby accelerating its pyrolysis and promoting flame propagation [144,145]. In contrast, the PS/PON/EG composite forms an intumescent char layer during combustion. As temperatures rise, this layer thickens and stabilizes through P–O–C bonds between PON’s phosphorus-containing structures, PS’s aromatic hydrocarbons, and graphite.

This results in a dense, continuous porous char layer that inhibits heat and gas transfer, providing condensed-phase flame retardancy. Additionally, PON’s pyrolysis releases phosphorus and nitrogen-containing compounds (e.g., PO/PO_2_, H_2_O, and NH_3_), which quench free radicals and dilute combustible gases, acting as gas-phase flame retardants [144,146,147]. The PON/EG system effectively disrupts the combustion cycle, enhancing PS’s flame retardancy. Similar mechanisms were observed by Wang and Bai [119], who noted a char layer of graphite flakes bonded with aromatic structures, and Zou et al. [148], who highlighted the role of phosphorus and nitrogen in quenching radicals and diluting gases. The flame-retardant performance of the synergistic PON/EG system is consistent with these observations, as it combines both condensed-phase and gas-phase mechanisms to achieve efficient flame suppression.

**Figure 13:**
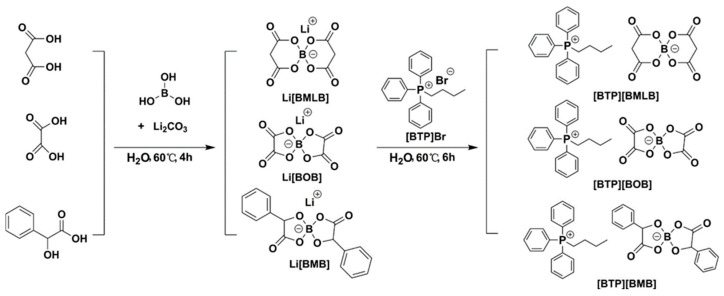
The preparation of butyltriphenylphosphine-based chelate borates, reprinted with permission from [116], Elsevier, Copyright 2023.

Guo et al. [116] developed a self-expanding intumescent flame retardant consisting of ammonium polyphosphate and expandable graphite. This formulation was then combined with three different chelate borates derived from butyltriphenylphosphine to evaluate their effect on the flame-retardant properties of polystyrene. This three-component flame-retardant system was formulated by integrating self-expanding intumescent flame retardants (IFRs) with ionic liquids (ILs) to examine the effect of chelate borates on the flame-retardant efficiency of IFRs, as illustrated in Figure 13. The flame-retardant performance of polystyrene composites containing this system was evaluated using LOI, UL-94, and CC tests as summarized in Table 3. The underlying flame-retardant mechanism was also explored. Among the chelate borates, [BTP][BMB] significantly enhanced flame retardancy, achieving a V-0 rating, an LOI of 27.0  ±  0.3%, and reduced heat release and smoke production with a 17 wt% total flame-retardant loading. Analysis of combustion residues using SEM and FTIR, along with pyrolysis gas investigation via TG-FTIR, provided insights into the flame-retardant mechanism. Additionally, rheological studies indicated that the enhancement in flame retardancy was associated with the temperature at which a crosslinked network formed due to expandable graphite, and this process was differentially influenced by the presence of chelate borates. The study proposes a flame-retarding mechanism based on rapid response to flame exposure.

Wang et al. successfully developed halogen-free flame-retardant PS foams using supercritical carbon dioxide (SC-CO_2_) as the foaming agent. The study explored the effects EG and MP on foamability, fire resistance, thermal decomposition, and mechanical properties. The flame retardants were found to release inert gases and promote char formation, creating a thick, protective char layer that significantly reduces heat release. Adding flame-retardant plasticizers such as triphenyl phosphate (TPP) or HPCTP improved foamability and fire performance. These additives release active phosphorus species and phenoxyl radicals, enhancing gas-phase flame retardancy. As a result, PS foams with 25 wt% MP/EG achieved HF1 and V-0 ratings, with flame retardancy improvement of LOI, PHRR, THR, and TTI with values of 29.6%, 168 kW/m^2^, 18.63 MJ/m^2^, and 14 s, respectively, as shown in Table 3 [117].

**Figure 14:**
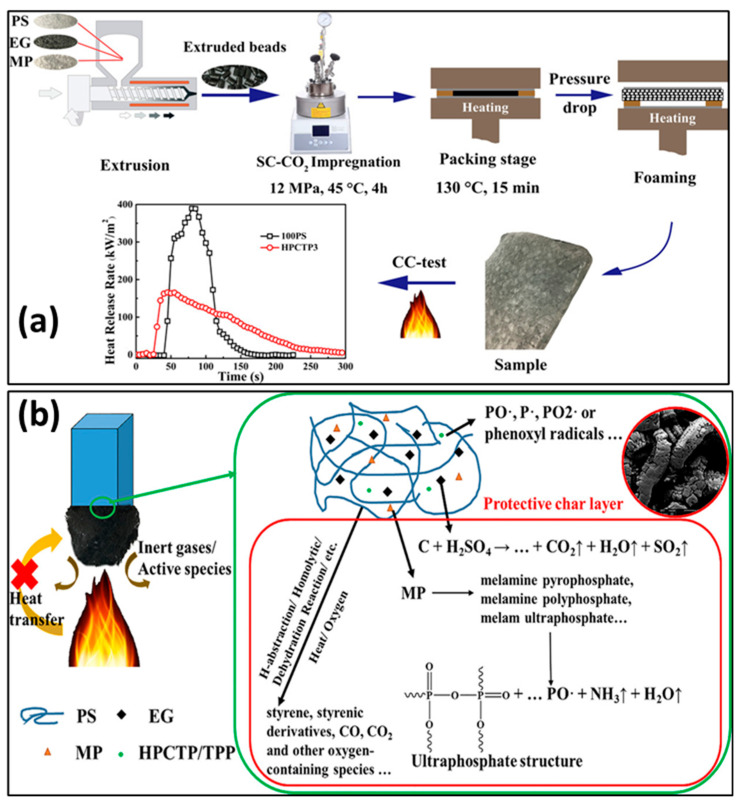
(**a**) Synthesis procedure of flame-retardant PS and (**b**) proposed flame-retardant mechanism for PS foam, reprinted with permission from [117], under the terms of the ACS AuthorChoice License.

Figure 14a indicates the synthesis procedure of the flame-retardant PS foams, by premixing PS with flame retardants and extruding them using a twin-screw extruder at 180 °C and 150 rpm. The extruded pellets were saturated with supercritical CO_2_ (SC-CO_2_) at 45 °C and 12 MPa for 4 h. The saturated pellets were then foamed via hot-press molding at 20 MPa and a specific foaming temperature. After 15 min, rapid pressure release yielded the flame-retardant foams. The flame-retardant mechanism of PS foams involves the combined action of EG and MP (Figure 14b).

When heated, EG undergoes a redox reaction (>190 °C) between sulfuric acid and graphite, forming worm-like expanded graphite. MP decomposes through complex reactions (condensation, chain scission, and crosslinking), producing intermediates such as melamine pyrophosphate, melamine polyphosphate, and non-flammable gases (e.g., water, ammonia). High-viscosity phosphate products from MP coat the expanded graphite, preventing oxidation and forming a thick, protective char layer with excellent barrier properties. Inert gases (e.g., SO_2_, CO_2_, H_2_O, and NH_3_) released by EG and MP dilute fuel and oxygen concentrations. Additionally, TPP and HPCTP act as synergists, decomposing into active phosphorus species (e.g., PO, P, and PO_2_) and phenoxyl radicals [149], which quench flammable free radicals (e.g., H, OH) and suppress combustion. The synergistic effects of gas-phase and condensed-phase flame-retardant greatly improve the flame resistance of PS foams [117].

**Figure 15:**
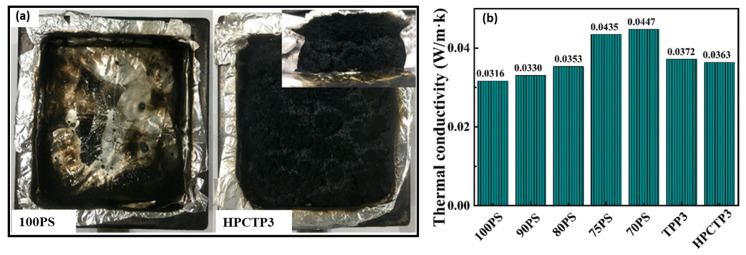
(**a**) The residual char of 100PS and HPCTP3 foams following cone calorimetry testing, and (**b**) the thermal conductivity values of various flame-retardant PS foams, reprinted with premissionfrom [117], American Chemical Society, under the terms of the ACS AuthorChoice License.

Figure 15a shows the residual char of 100PS and HPCTP3 foams following cone calorimetry testing. The 100PS foam yields negligible residue, whereas the HPCTP3 foam retains approximately 21 wt% of char. Upon heating, EG generates worm-like structures, and MP decomposes into high-viscosity phosphate species that reinforce the interlayer cohesion of graphite. This results in the formation of a dense and robust char layer that serves as a physical barrier, effectively impeding the transfer of heat, oxygen, and volatile decomposition products. This condensed-phase flame-retardant mechanism notably improves the fire resistance of polystyrene foams. Additionally, Figure 15b presents the thermal conductivity measurements of the flame-retardant PS foams. As the MP/EG content increases, the thermal conductivity rises due to two factors: higher foam density and the formation of heat-conducting paths by EG particles, which have greater thermal conductivity than PS. The thermal conductivity of 75 PS foam is 0.0435 W/(m·K). However, adding TPP or HPCTP improves foaming performance, reducing foam density and lowering thermal conductivity to 0.0372 W/(m·K) for TPP3 and 0.0363 W/(m·K) for HPCTP3 foams.

**Figure 16:**
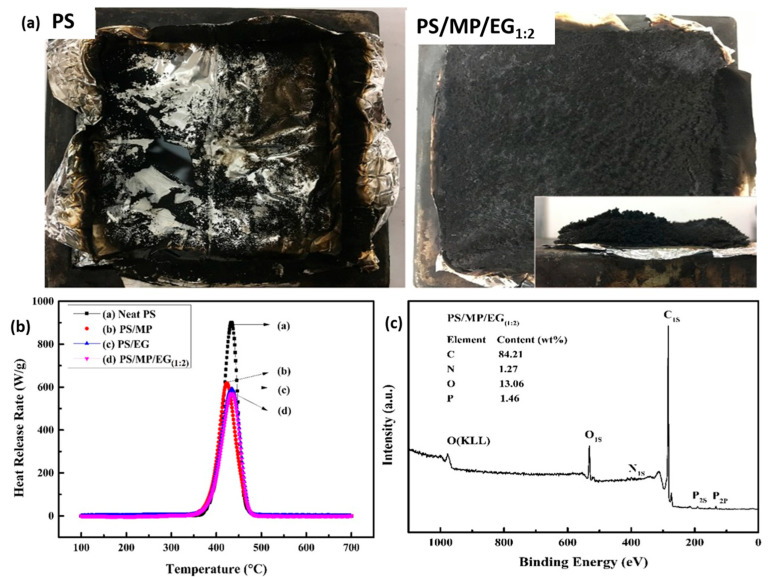
(**a**) HRR curves of PS/MP/EG composites of the MCC. (**b**) The residues of PS and PS/MP/EG_(1:2)_ composite after cone calorimetric test. (**c**) XPS spectra of the heat-treated PS/MP/EG_(1:2)_ composite at 700 °C, reprinted with permission from [119], John Wiley & Sons, Inc. Copyright © 2017 John Wiley & Sons. License No. 6034200556800.

Wang and Bai [119] successfully synthesized a halogen-free flame-retardant PS composites by incorporating MP and EG, and comprehensively assessed their thermal degradation behavior and fire-resistant properties. The results demonstrate a synergistic interaction between EG and MP in the PS matrix, leading to improved char formation. Notably, the PS/MP/EG_(1:2)_ composite attained a LOI of 28.0% and achieved a UL-94 V-0 classification at a thickness of 1.6 mm. The introduction of EG and MP significantly increased the mass retention of PS composites at high temperatures (800 °C) under air. MCC and CC tests revealed a substantial reduction HRR and THR for the PS/MP/EG_(1:2)_ composite, ascribed to the development of a dense, protective char layer with superior barrier functionality. X-ray photoelectron spectroscopy (XPS) analysis of the char residue confirmed the presence of stable structures containing P−O−C bonds. Additionally, while the tensile and flexural strengths of the PS composites decreased compared to neat PS, the impact strength of the PS/MP/EG_(1:2)_ composite increased by 44.2%. Figure 16a illustrates the MCC result, PS exhibited a high peak HRR of 956.7 W/g, which significantly decreased as a result of flame-retardant integration. The combination of MP and EG was particularly effective, reducing peak HRR by 40.4%. The PS/MP/EG_(1:2)_ composite showed the lowest THR, decreasing from 37.2 to 28.2 kJ/g, while HRC dropped by 41.3% from 980.0 to 575.0 J/g·K. This substantial reduction highlights the enhanced flame retardancy, attributed to the release of inert gases that dilute flammable components and the vesicular structure of EG, which prolongs pyrolysis product diffusion. These mechanisms indicate a synergistic effect between MP and EG in improving PS flame resistance.

Figure 16b displays the residues of PS and the PS/MP/EG_(1:2)_ composite following the cone calorimetry test. Notably, PS leaves no residue after combustion. In contrast, the PS/MP/EG_(1:2)_ composite forms a thick char layer, demonstrating significant barrier properties. This dense char layer acts as an effective insulator, blocking heat, air, and pyrolysis products, thereby greatly enhancing the material’s flame retardancy. Additionally, the protective effect of the char layer prevents the underlying matrix from burning completely, leaving a portion of it unburned. Figure 16c indicates that the XPS analysis of the PS/MP/EG_(1:2)_ composite char residue revealed its elemental composition (84.21 wt% C, 13.06 wt% O, 1.46 wt% P, and 1.27 wt% N) and chemical bonds, including C–H, C–C, C–O–P, and C=O [150,151]. The presence of phosphorus and nitrogen facilitated the formation of stable structures, such as C–O–P, which improved char retention at high temperatures. This result, consistent with TGA findings, indicates that greater char residue formation enhances thermal insulation properties.

Moreover, the mechanical properties of the composites show that, relative to neat PS, the tensile and flexural strengths of PS/EG and PS/MP/EG (1:2) reach 45.37 and 92.49 MPa, and 44.83 and 90.66 MPa, respectively, corresponding to reductions of 14.7% and 15.1%, and 15.7% and 16.8%. The decrease in tensile and flexural strength is attributed to the poor compatibility between PS and flame retardants, with MP exerting a more pronounced effect. EG, characterized by its flat, multilayer structure with an average particle size of approximately 200 µm, enhances impact strength due to its energy absorption mechanism during fracture. The impact strength of PS/EG and PS/MP/EG_(1:2)_ increased by 50.5% and 44.2%, respectively, owing to the pull-out and breakage of EG flakes. Overall, the incorporation of flame retardants significantly enhances the flame retardancy of PS composites while causing only minimal degradation in mechanical properties. Furthermore, TGA analysis under N_2_ atmosphere revealed that pure PS decomposes rapidly between 379.4 °C and 450 °C, while flame-retardant composites show varying initial decomposition temperatures: PS/MP and PS/MP/EG_(1:2)_ decompose earlier due to MP, whereas PS/EG decomposes at a higher temperature due to expanded graphite formation. All flame-retardant composites form stable char residues above 450–500 °C, with PS/EG exhibiting minimal weight loss up to 800 °C. Residue yields at 800 °C were 4.25 wt% (PS/MP), 15.07 wt% (PS/EG), and 12.04 wt% (PS/MP/EG_(1:2)_) [119].

The comparative analysis of various graphene-based functionalization approaches and flame-retardant systems for polystyrene reveals distinct advantages and inherent trade-offs. Primarily, there is an existing literature gap on graphene-based functionalization and hybrid flame-retardant systems for polystyrene. Non-functionalized GO, such as the variant employed by Sabet et al., [93] shows only small improvement in thermal stability and LOI values, highlighting fundamental limitations related to poor interfacial adhesion, and graphene agglomeration. The limited effectiveness reported, such as an LOI increase from 18% to merely 18.8% at 3 wt.%, shows a clear need for functionalization or hybrid strategies to enhance graphene-polymer interactions. However, many studies attribute these minor enhancements to generic barrier effects, ignoring the structural or morphological factors that might govern such performance.

In comparison, covalent functionalization of graphene through phosphorus, demonstrated by Dai et al. [113] enhanced the interfacial compatibility and dispersion quality. This resulted in substantial increases in LOI (up to 25%) and a significant decrease in heat release capacities (39% reduction). However, covalent approaches also include several trade-offs such as a multi-step process, high costs, scalability challenges, and risk of degrading graphene’s intrinsic properties. The existing studies often sidestep these trade-offs or to clearly quantify the comparative advantages of covalent versus non-covalent functionalization strategies. Similarly, non-covalent or physically blended flame-retardant systems can offer synergistic effects via both condensed and gas-phase mechanisms. However, this is often at the cost of reduced mechanical properties. This trade-off remains insufficiently examined, highlighting a key gap in balancing fire resistance with long-term material durability. Furthermore, a comparative and systematic assessment of such approaches needs to be explored and benchmarked against alternative functionalization strategies. Additionally, fewer studies explicitly address the practical challenges of the high thermal conductivity of graphene, especially in building insulation applications.

## 5. Outlook on Graphene-Based Flame Retardants for Polystyrene Applications

The transition of graphene-based flame retardants from laboratory scale to industrial-scale applications is limited by several critical challenges which need to be resolved. Foremost among these are issues of dispersion quality, scalability of synthesis, cost-efficiency, and environmental sustainability. Achieving uniform dispersion of graphene nanosheets is still a major challenge. The agglomeration of graphene because of its strong intermolecular attractions often results in non-uniform mechanical properties and inconsistent flame-retardant performance. More studies are needed to develop advanced dispersion strategies, such as high-throughput ultrasonic-assisted liquid-phase exfoliation combined with tailored surfactants or bio-inspired dispersing agents as recently demonstrated by Kaur et al. to mitigate agglomeration at large scales [152]. Furthermore, high thermal conductivity of graphene is also a challenge for its applications in insulating materials. To cope with this issue, innovative solutions are required, such as insulating aerogel coatings [153], which provide flame retardancy by creating an insulating barrier without significantly impacting the thermal conductivity.

The incompatibility of hydrophilic graphene derivatives with hydrophobic polystyrene is yet another significant challenge. Even though covalent functionalization works well for this problem, it compromises the inherent qualities of graphene by adding complexity and structural defects. Other options might result from non-covalent functionalization with amphiphilic molecules, polymer grafting, or mussel-inspired polydopamine coatings (as shown by Liu et al., [154]). Furthermore, another challenge of using graphene as a flame retardant in PS is the high filler loading requirements, such as 25 wt.%. to form an effective char barrier [94]. Nevertheless, such high loadings have an adverse effect on mechanical strength, ductility, and processability of the polymer. Future studies should focus on multifunctional graphene-based nanohybrids that demonstrate synergistic flame retardancy at lower loadings. For instance, coupling graphene with phosphorus- or nitrogen-containing compounds such as ammonium polyphosphate, melamine phosphate, or DOPO has shown the potential to significantly enhance flame resistance while reducing the total additive content to below 3 wt.% [113]. These hybrids can promote intumescent gas-phase radical quenching and char formation, thereby compensating for the lower graphene concentration. Furthermore, scalable and cost-effective graphene production is also a key challenge. Recent developments, such as the graphitization of petroleum-derived waste streams (vacuum residues and asphaltene), yielding high-quality graphene at a significantly reduced cost [155], highlight viable pathways toward scalable, environmentally benign production methods of graphene. Future research should focus on optimizing these scalable methods to promote large-scale production and applications of graphene. Concurrently, computational techniques using artificial intelligence (AI) should be utilized for the identification of functionalization methods and synergistic combinations as demonstrated by predictive modeling material discovery [156].

In essence, the practical utilization of graphene based flame-retardant polystyrene composites requires coordinated interdisciplinary efforts. By combining the expertise in nanoscale functionalization, composite processing, and environmental assessments we can bridge the gap between laboratory research and commercial utilization.

## 6. Conclusions

Graphene-based flame retardants gained significant attention as an effective approach to overcoming the inherent flammability and thermal limitations of polystyrene. Graphene offers thermal stability, barrier properties, mechanical reinforcement, and environmental friendliness. This review explored synthesis methods, flame-retardant mechanisms, and recent advancements in graphene-based systems for PS applications, emphasizing the potential of functionalized graphene derivatives to enhance dispersion, interfacial compatibility, and multifunctionality. Despite these significant advantages, challenges such as agglomeration, high thermal conductivity, scalability, cost-effectiveness, and maintaining long-term performance hinder their large-scale adoption, particularly in energy-efficient building materials. To address these challenges, future research should prioritize advanced surface functionalization techniques, the design of hybrid flame-retardant systems to maximize synergistic effects, and the optimization of graphene content to optimize the trade-off between flame-retardant performance and insulation effectiveness. Additionally, advanced dispersion techniques, sustainable manufacturing methods, and comprehensive property evaluations will be crucial to ensure these materials meet industrial requirements. By addressing these challenges, graphene-based flame retardants can be further developed into highly efficient, multifunctional, and sustainable materials. Their integration into circular economy principles and their potential for combining flame retardancy with additional functionalities, such as electrical conductivity or energy storage, offer exciting opportunities for advancing material science and sustainability, ultimately contributing to significant improvements in fire safety and energy efficiency for PS-based applications.

## Figures and Tables

**Table 1:** UL-94 tests classification table according to IEC 60695-11-10 [49]. © 2020 by the authors; licensed under CC BY 4.0 [43].

S/N	Evaluation Standards	Fire Classification		
		V-0	V-1	V-2
1	Number of samples	5	5	5
2	Maximum flame burning time (s) per sample after flame removal	10	30	30
3	Maximum total flaming time (s) for 5 samples (10 ignitions)	50	250	250
4	Maximum afterglow time (s) per sample after second flame application	30	60	60
5	Flames or afterglow reaching the holding clamp	No	No	No
6	Dripping of flaming particles igniting cotton	No	No	Yes
7	Burning time (s)	2	2	2

**Table 2:** Overview of top-down and bottom-up approaches for graphene growth mechanisms, © 2016 by the authors; licensed under CC [73].

Method	Lateral	Descriptions	Thickness	Advantage	Disadvantage
Micromechanical exfoliation	μm to cm	This technique involves using adhesive tape to strip graphene layers from a graphite source.	Few layers	Pristine, unaltered graphene sheets of substantial size.	Extremely limited production scale, rendering it unsuitable for large-scale industrial manufacturing.
Electrochemical exfoliation	500–700 nm	This technique utilizes an electric field applied to graphite within an electrolyte solution to separate graphene layers.	Single to few layers	Potential for large-scale production and yields functionalized graphene with high electrical conductivity.	High cost associated with ionic liquids.
Direct sonication of graphene	μm	This technique uses ultrasonic waves to exfoliate graphite into graphene within a solvent medium. It is simple but yields mixed-quality graphene and is better for small-scale use.	Includes both monolayer and multilayer structures.	Cost-effective and pristine graphene.	Produces low yield and quality graphene, making it ideal for small-scale or research use.
Reduction in carbon monoxide	Sub-μm	GO can be produced by oxidizing graphite and subsequently reducing it to graphene using chemical or thermal reduction methods. This approach is ideal for generating graphene that retains functional groups.	Multiple layers	Un-oxidized sheets	Contamination with α-Al_2_S and α-Al_2_O_3_
Epitaxial growth on SIC	Up to cm size	This involves heating silicon carbide to evaporate silicon, forming high-quality graphene on the surface. It is ideal for electronics but requires high temperatures.	Few layers	Very large area of pure graphene	Very small scale, require expensive equipment, and the graphene remains substrate-bound.
CVD	Very large (cm)	CVD entails the deposition of a carbon-based gas onto a substrate under high temperatures, where methane is commonly used as a precursor gas.	Few layers	Large size and area; high-quality graphene.	Small production scale and require high temperature.
Unzipping of carbon nanotubes	Few μm long nano ribbons	This technique involves chemically or physically splitting nanotubes into graphene nanoribbons or sheets. It produces tailored graphene with defined edges, useful for electronics.	Multiple layers	Size is determined by the initial nanotube dimensions.	Expensive and oxidized graphene. Additionally, it requires precise control for consistent quality.

**Table 3:** Summary of different graphene-based materials used for flame-retardant applications.

Material	Polymer	Material Loading (wt%)	LOI (%)	UL-94	PHRR (kW/m^2^)	THR (MJ/m^2^)	TTI (s)	Ref.
G	PA6	2	__	__	1257	133.1	79	[84]
GNS-CoO_4_					1282	141.4	81	
GNS-NiO					1105	130	75	
GO	PS	2	18.9	__	384	131	36	[92]
GO	PS	2	18.8	__	385	130	37	[93]
GO	PVA	5	__	__	133	38	45	[24]
PS-S-GRF	PS	30	24	HB	479	89	23	[94]
ZnAl-G-PCL	PS	15	20.01	__	__	__	5	[5]
FGO	PP	2	__	__	739	98.7	33	[95]
EPS-44%DG/EG	EPS	44	36.0	V-0	138.2	12.6	3	[96]
DPP-GO	EP	4	25.2	V-0	301.9	13.2	3.2	[97]
P-N-rGO	EP	5	30.5	V-1	785.7	57.7	56	[98]
G-DOPO	EP	5	__	HB	538	36.5	32	[32]
GO-DOPO	TPU	2	__	__	362	48	__	[99]
APP-CFA-G	PP	25	32	V-0	140	90.4	35	[100]
G-MDP-TiO_2_NP	ABS	30	__	HB	720	75	35	[101]
GO-DOPO-V	EP	2	__	__	1552.78	78.97	__	[102]
DGO	EPS	20	29	V-0	304.6	39.9	31	[103]
Mo5/PN-rGO	ABS	1	__	__	362	99	59	[104]
EG	EPS	33	22	No	144.90	28.22	__	[105]
RP-EG (1:2)			25	V-0	__	__	2.5/4.5	
RP-EG (1:1)			26.9	V-0	180.67	61.01	1.5/7.5	
RP-EG (2:1)			26.1	V-1	__	__	7.5/3	
FGO–AHP	PS	5	__	__	639	34.9	__	[106]
PAUCG	EPS	__	48.0	V-0	36.5	4.56	22.0	[107]
SD_8_ + graphene	PR	0.5	__	__	31.5	10.36	288	[108]
FR-fGO	EP	1	29.2	V-1	927.23	70	__	[109]
PON-EG	PS	20	25.8	V-0	242.0	90.6	34	[110]
PON-GN			23.5	V-1	321.8	90.7	42	
PSS@GNS	PS	1	__	__	1036.2	77.6	45	[111]
		2			1007.6	65.0	51	
		4			763.7	62.1	56	
ATG	EPS	20	35.5	V-0	201.4	9.0	227	[112]
GO	PS	1	21	__	441	24.2	__	[113]
FGO		1	22.5		445	23.4		
		2	24.0		438	22.7		
		3	25.0		436	21.4		
LDH-DBP-5 wt%/GO-DDA-1.0 wt%	PS	6	20	__	456	124	65	[114]
TGO	PS	8	__	__	452	69	17	[115]
PS1	PS	17	24.1	V-2	334.2	79.0	28	[116]
PS2		17	24.8	V-2	351.6	79.2	34	
PS3		17	24.8	V-2	415.7	85.2	33	
PS4		17	27.0	V-0	319.6	74.6	31	
HPCTP/MP/EG	PS	25	29.6	HF1 and V-0	169	18.63	14	[117]
EG15/MRP5	HIPS	20	26.8	V-0	191	59	54	[118]
FGO2.0	PS	30	__	__	514 W/g	26.9 kJ/g	__	[38]
RPEG	EPS	33	26.9	V-0	180.67	61.01	__	[105]
PSS@GNS	PS	4	__	__	763.7	62.1	56	[111]
MP/EG_(1:2)_	PS	20	28	V-0	209.7	104.9	44	[119]

EPS = expandable polystyrene, EG = expandable graphite, DPP = 10-dihydro-9-oxa-10-phosphaphenanthrene-10-oxide-g (2,3-epoxypropoxy) propyltrimethoxysilane, APP = ammoniumpolyphosphate, ABS = Poly (acrylonitrile-butadiene-styrene), MDP = maleate diphosphate, TPU = thermoplastic polyurethane, PON = tetraphenyl piperazine-1,4-diyldiphosphonate, ATG = phenolic epoxy resin, ammonium polyphosphate and tannic acid functionalized graphene, PSS@GNS = polystyrene sulfonate functionalized graphene nanosheets, TGO = thermally reduced graphene oxides, RP = red phosphorus, HPCTP = hexaphenoxycyclotriphosphazen, MP = melamine phosphate, HIPS = high-impact polystyrene, MRP = microencapsulated red phosphorus, DG = polysiloxane-based flame-retardant adhesive, and MP = melamine phosphate.

## Data Availability

Not applicable.
